# Dopant‐Free Hole Transport Materials Afford Efficient and Stable Inorganic Perovskite Solar Cells and Modules

**DOI:** 10.1002/anie.202107774

**Published:** 2021-08-07

**Authors:** Cheng Liu, Cansu Igci, Yi Yang, Olga A. Syzgantseva, Maria A. Syzgantseva, Kasparas Rakstys, Hiroyuki Kanda, Naoyuki Shibayama, Bin Ding, Xianfu Zhang, Vygintas Jankauskas, Yong Ding, Songyuan Dai, Paul J. Dyson, Mohammad Khaja Nazeeruddin

**Affiliations:** ^1^ State Key Laboratory of Alternate Electrical Power System with Renewable Energy Sources North China Electric Power University Beijing 102206 P. R. China; ^2^ Group for Molecular Engineering of Functional Materials Institute of Chemical Sciences and Engineering EPFL VALAIS 1951 Sion Switzerland; ^3^ Department of Chemistry Lomonosov Moscow State University Moscow 119991 Russia; ^4^ Department of Organic Chemistry Kaunas University of Technology Kaunas 50254 Lithuania; ^5^ Department of Biomedical Engineering Toin University of Yokohama 1614 Kurogane, Aoba Yokohama Japan; ^6^ Institute of Chemical Physics Vilnius University Sauletekio al. 3 Vilnius 10257 Lithuania; ^7^ Department of Materials Science and Engineering City University of Hong Kong Kowloon Hong Kong

**Keywords:** defect passivation, dopant-free, hole transport materials, inorganic perovskites, perovskite solar cells

## Abstract

The emerging CsPbI_3_ perovskites are highly efficient and thermally stable materials for wide‐band gap perovskite solar cells (PSCs), but the doped hole transport materials (HTMs) accelerate the undesirable phase transition of CsPbI_3_ in ambient. Herein, a dopant‐free D‐π‐A type HTM named CI‐TTIN‐2F has been developed which overcomes this problem. The suitable optoelectronic properties and energy‐level alignment endow CI‐TTIN‐2F with excellent charge collection properties. Moreover, CI‐TTIN‐2F provides multisite defect‐healing effects on the defective sites of CsPbI_3_ surface. Inorganic CsPbI_3_ PSCs with CI‐TTIN‐2F HTM feature high efficiencies up to 15.9 %, along with 86 % efficiency retention after 1000 h under ambient conditions. Inorganic perovskite solar modules were also fabricated that exhibiting an efficiency of 11.0 % with a record area of 27 cm^2^. This work confirms that using efficient dopant‐free HTMs is an attractive strategy to stabilize inorganic PSCs for their future scale‐up.

## Graphical abstract

A dopant‐free D‐π‐A type HTM named CI‐TTIN‐2F has been developed which shows excellent charge collection properties and multisite defect‐healing effects. All‐inorganic CsPbI_3_ PSCs with CI‐TTIN‐2F HTM feature high efficiencies up to 15.9 %, along with 86 % efficiency retention after 1000 h under ambient conditions. All‐inorganic perovskite solar modules were also fabricated that exhibit an efficiency of 11.0 % with a record area of 27 cm^2^.

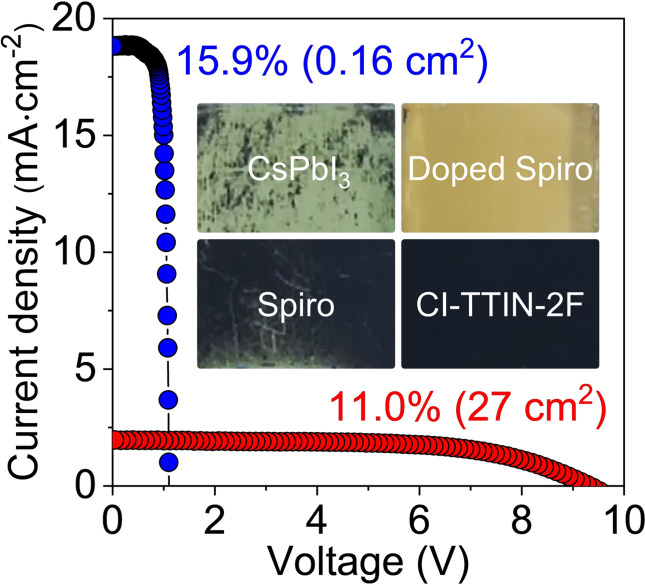

## Introduction

Hybrid organic‐inorganic perovskite solar cells (PSCs) have demonstrated remarkable progress in power conversion efficiencies (PCEs) from 3.8 % to 25.5 % in the past several years, showing their great potential in next‐generation low‐cost photovoltaic technology.^[1]^ However, the poor thermal stability of hybrid organic‐inorganic perovskite materials hinders their large‐scale commercialization.^[2]^ Thus, a significant part of the research focus has been shifted to the all‐inorganic perovskite structures without volatile organic components.^[3]^ Among the various inorganic monovalent cations, Cs^+^ is expected to be the most feasible candidate to substitute the organic cations for two reasons.^[4]^ The first relates to its larger size, which satisfies the geometrical constraints of the perovskite structure to establish the continuous array of corner‐sharing PbI_6_ octahedra.^[5]^ The second arises from the superb thermal stability of CsPbX_3_ (X=Cl, Br, and I) materials which may be compositionally stable even at temperatures exceeding 400 °C.^[6]^ So far, the efficiencies of all‐inorganic Pb‐halide PSCs have exceeded 20 % in an n‐i‐p structure, which was achieved by the tetragonal (β) CsPbI_3_ perovskite with an ideal band gap of 1.68 eV for photovoltaic application.^[7]^ Nevertheless, the imperfect Goldschmidt tolerance factor of black phase CsPbI_3_ determines that their PbI_6_ octahedra tends to rotate when catalyzed by H_2_O, which induces a rapid phase transition to the non‐photovoltaic yellow δ phase.^[8]^

Several strategies were reported to address this stability issue including surface energy tuning,[^5^, ^9^] additive engineering,^[10]^ and interfacial passivation.^[11]^ However, the implementation of these strategies for improving stability is limited due to the potential impact on device performance. For instance, the amount of the additive is normally low in order to maintain crystallinity and charge mobility of the perovskite film.^[12]^ Similarly, the thickness of the surface passivation layer must be a few nanometers to maintain charge extraction from the active layers to the charge transport layers.^[13]^ For these reasons, highly hydrophobic hole transport materials (HTMs) have received attention, as they have the potential to block the moisture‐driven phase transition of black phase CsPbI_3_.^[14]^

At present, state‐of‐the‐art CsPbI_3_ PSCs still use 2,2′,7,7′‐tetrakis(*N*,*N*‐di‐*p*‐methoxyphenyl‐amino)‐9,9′‐spi‐robifluorene (spiro‐OMeTAD) doped with lithium bis(trifluoromethane)sulfonamide (LiTFSI), cobalt complex and 4‐*tert*‐butyl pyridine (TBP) additives as the HTM.[^7b^, ^15^] However, we observed very fast phase transitions (Figure S19) in the black phase CsPbI_3_ films covered with standard doped spiro‐OMeTAD layers, with these transitions being even faster than that of bare CsPbI_3_ films under the same conditions. Hence, the hygroscopic nature of the dopants eliminates the advantage of the hydrophobicity of the upper hole transport layers and significantly accelerates the phase transition of CsPbI_3_ leading to device degradation.^[16]^ Therefore, developing dopant‐free, efficient, and stable HTMs is highly desirable and could lead to practical applications of inorganic perovskites in PSCs.

Herein, we described the design and synthesis of a dopant‐free D‐π‐A type HTM, namely CI‐TTIN‐2F, employing a triazatruxene (TAT) as the electron‐rich donor, alkylated terthiophene as π‐bridges, and a fluorinated Lewis base as the electron‐deficient acceptor. Benefiting from intramolecular charge transfer (ICT) and strong dipolar intermolecular interactions, CI‐TTIN‐2F shows excellent optoelectronic properties, ideal energy‐level alignment, and good charge collection properties. In addition, joint experimental and theoretical studies suggest that CI‐TTIN‐2F provides multisite defect‐healing effects on the surface of the CsPbI_3_ films due to the presence of various heteroatoms (N, O, S, F) in the HTM structure, which effectively reduces the trap densities and charge recombination at the interface. As a dopant‐free HTM for the all‐inorganic CsPbI_3_ PSCs, CI‐TTIN‐2F impressively delivers high PCEs of 15.9 % on small‐area cells and 11.0 % on modules with a record area of 27 cm^2^. Furthermore, the device employing CI‐TTIN‐2F maintains over 86 % of its initial performance for 1000 h after storage in ambient conditions.

## Results and Discussion

**Figure 1:**
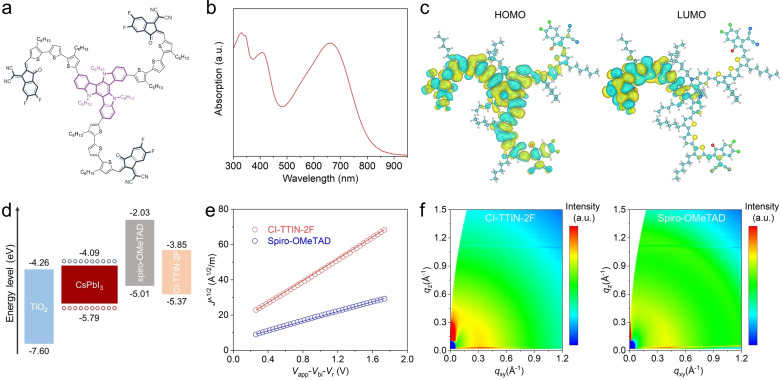
a) Chemical structure of the CI‐TTIN‐2F molecule. b) UV‐vis absorption spectra of CI‐TTIN‐2F in dilute tetrachloroethane solution. c) Spatial distribution of HOMO and LUMO orbitals in CI‐TTIN‐2F. C, O, N, H, F and S atoms are colored in cyan, red, blue, white, green and yellow, respectively. d) Molecular energy level alignments. e) The hole injection characteristics measured by the SCLC method of dopant‐free HTMs. f) 2D GIWAXS patterns of pristine hole transport layers.

Figure 1 a depicts the chemical structure of CI‐TTIN‐2F HTMs. The synthetic routes and the synthetic details are given in the Supplemental Information (Figures S1–S3). A planar nitrogen‐containing triazatruxene (TAT) core with alkyl chains was employed as the donor (D) due to its excellent charge transporting and strong π‐π stacking ability.^[17]^ The alkylated terthiophene conjugated arms, 3,3′′‐dihexyl‐2,2′:5′,2′′‐terthiophene, were modulated as π‐bridge to increase hole‐mobility properties of the molecule by increasing the double‐bond character.^[18]^ A strong Lewis base electron‐withdrawing acceptor (A) unit, 2‐(5,6‐difluoro‐3‐oxo‐2,3‐dihydro‐1*H*‐inden‐1‐ylidene)malononitrile (IN‐2F), was selected based on D‐π‐A type architecture to decrease the LUMO levels without causing strong steric hindrance, enhance inter/intramolecular non‐covalent interactions, and increase hydrophobicity, leading to long‐term stability. The expected structure of CI‐TTIN‐2F was confirmed by MALDI‐TOF mass spectrometry (Figure S4). To evaluate the thermal properties of CI‐TTIN‐2F thermogravimetric analysis (TGA) was carried out under a nitrogen atmosphere (Figure S5). The decomposition temperature (*T*_dec_) corresponding to a weight loss of 5 % occurs above 300 °C, indicating that CI‐TTIN‐2F has sufficient thermal stability for application as a HTM in PSCs. Moreover, CI‐TTIN‐2F is soluble in tetrachloroethane, and reasonably soluble in tetrahydrofuran and dichloromethane, allowing solution processing.

The optical properties of CI‐TTIN‐2F were investigated by ultraviolet‐visible (UV/Vis) spectroscopy in the solid state and in tetrachloroethane solution (Figure 1 b). The UV‐vis spectra of the CI‐TTIN‐2F film and solution show multimodal absorption (Figure S6). Absorption bands were observed between 350 and 500 nm, which may be attributed to the localized aromatic π‐π* transition of the D‐π‐A structure. More importantly, an additional NIR absorption peak centered at around 660 nm indicates ultra‐strong intermolecular charge transfer from the TAT electron‐donating unit to the IN‐2F electron‐withdrawing group. Note that the position of the ICT band is slightly red‐shifted as the solution transitions to the solid‐state, which is characteristic of many organic semiconductors.^[19]^ The optical energy band gap (*E*_g_) estimated from the onset of the absorption peak is determined to be 1.52 eV for CI‐TTIN‐2F, which would increase the intrinsic carrier concentration through the thermal population in the conduction band.

To investigate the highest occupied molecular orbital (HOMO) energy level (*E*_HOMO_) of CI‐TTIN‐2F, electrochemical cyclic voltammetry (CV) was first performed with a standard three‐electrode configuration (Figure S7). The material was tested in tetrahydrofuran containing 0.1 M *n*‐Bu_4_NPF_6_ as a supporting electrolyte, and the oxidation potential was calibrated against an internal ferrocene standard. The *E*_HOMO_ value was calculated as −5.37 eV vs. vacuum for CI‐TTIN‐2F. The HOMO level is well aligned with the valence band energy level of the CsPbI_3_ inorganic perovskite so that the photogenerated charge carriers should be efficiently transferred at the interface.^[20]^ The lowest unoccupied molecular orbital (LUMO) energy levels were calculated to be −3.85 eV by subtracting the optical band gap and HOMO energy level. These results are consistent with the trend estimated from density functional theory (DFT) calculations (Table S1 and S2). The molecular structures were optimized by using PBE‐D3 density functional. The localization and energies of the frontier molecular levels of CI‐TTIN‐2F for the HOMO are delocalized primarily in the TAT core and spread along the entire molecule, while LUMO are localized in the acceptor moiety and π‐bridge of the HTM (Figure 1 c). Figure 1 d shows the schematic of the energy band diagram. The energy bands of the CsPbI_3_ perovskite were characterized by ultraviolet photoelectron spectroscopy (UPS) and UV‐vis spectroscopy as shown in Figure S8. The energy level alignment implies that the fluorinated IN‐2F acceptor unit with stronger electron‐withdrawing properties endows CI‐TTIN‐2F with much deeper HOMO energy levels by 0.40 eV compared to spiro‐OMeTAD. The lower HOMO of CI‐TTIN‐2F is expected to ensure more efficient interfacial hole‐transport kinetics and improve the open‐circuit voltage (*V*_OC_) for CsPbI_3_ PSCs.^[21]^

The space‐charge limited current (SCLC) measurements were carried out on the hole‐only devices to evaluate the charge transport properties of CI‐TTIN‐2F, and the hole mobilities were extracted following the Mott‐Gurney Law (Figure 1 e). Encouragingly, with the D‐π‐A molecular structure, CI‐TTIN‐2F exhibits high hole mobility of 3.7×10^−4^ cm^2^ V^−1^ s^−1^, significantly higher than that of spiro‐OMeTAD (6.1×10^−5^ cm^2^ V^−1^ s^−1^), attributed to the higher degree of conjugation and better intermolecular interactions.^[22]^ Grazing incidence wide‐angle X‐ray scattering (GIWAXS) was performed to investigate the molecular organization in the HTM films (Figure 1 f). The pole plots of the azimuth angle integrated around *q*_z_=3.5 Å^−1^ (Figure S9) indicate that both CI‐TTIN‐2F and spiro‐OMeTAD possess multiple orientations in the films. Besides the face‐on orientation, CI‐TTIN‐2F exhibits edge‐on stacking that favor charge transport.^[17]^

**Figure 2:**
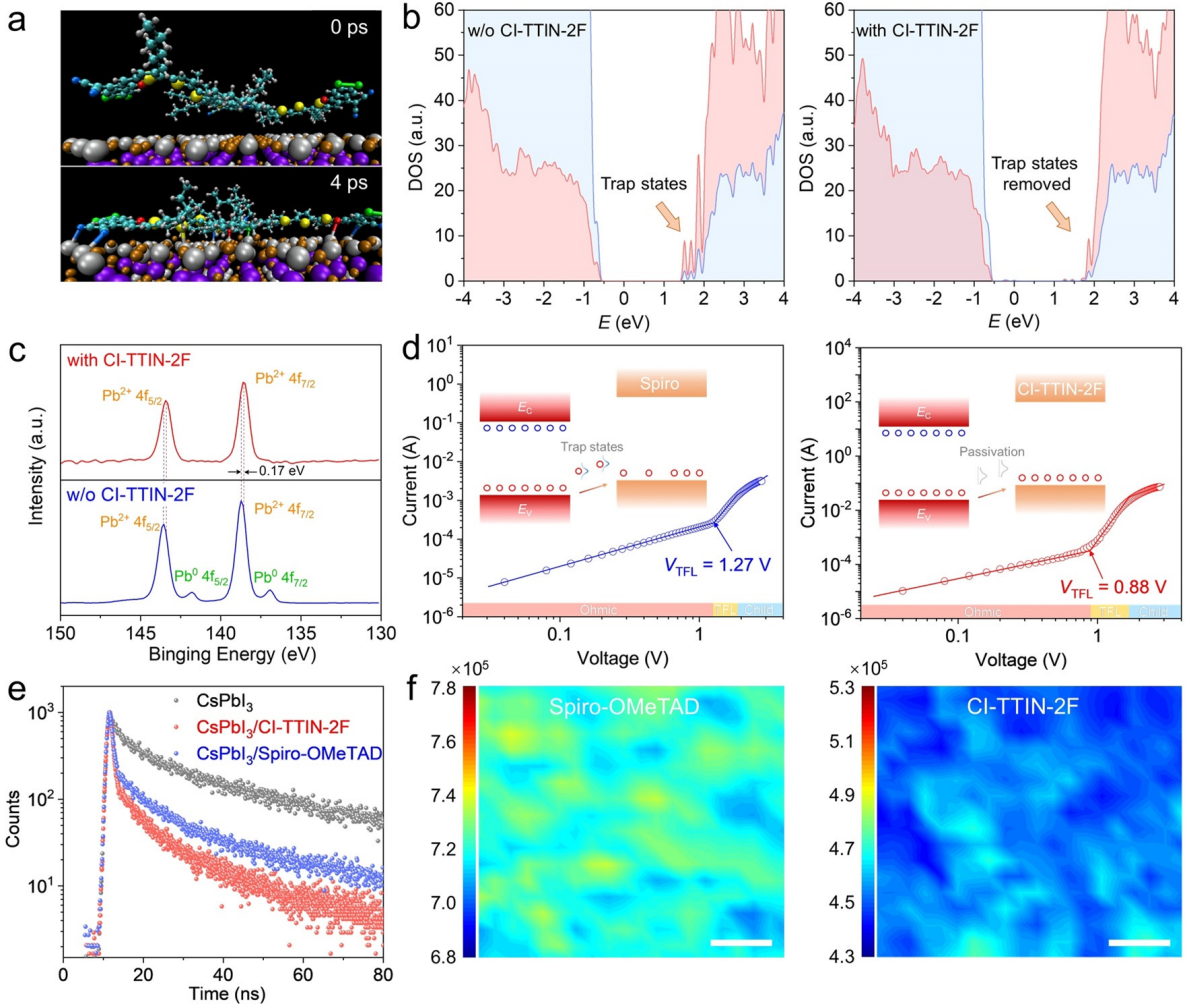
a) Molecular dynamics calculations of the CI‐TTIN‐2F molecule deposited on top of the slab of CsPbI_3_ perovskite. b) Electronic structure of the PbI_2_‐terminated surface without and with CI‐TTIN‐2F HTMs, calculated using PBE0 density functional. c) The XPS of Pb 4f in the CsPbI_3_ with and without CI‐TTIN‐2F films. d) The space charge limited current versus voltage of hole‐only devices fabricated with spiro and CI‐TTIN‐2F HTMs. e) TRPL spectra and f) PL intensity maps of CsPbI_3_ films on glass substrate capping with different HTMs. Scale bar, 1 μm.

To investigate the potential interactions occurring between the CI‐TTIN‐2F HTM and the perovskite surface, Born Oppenheimer molecular dynamics (MD) calculations of CI‐TTIN‐2F deposited on top of a slab of tetragonal β‐CsPbI_3_ perovskite were performed (Figure 2 a, movie in SI). The MD simulations show that CI‐TTIN‐2F can form Pb contacts of different types with the perovskite surface, specifically: i) The CN groups of the acceptor units interact with the Pb^2+^ cations of the surface, in particular forming relatively short Pb⋅⋅⋅N contacts of 2.8–3.0 Å (Figure S10), evidencing strong coordination interactions. ii) The F atoms of the acceptor can interact weakly with the surface with average Pb‐F distances >4 Å (Figure S11). This is partially due to the face‐on configuration of CI‐TTIN‐2F CN groups within 2FIN which coordinate with the perovskite surface, as malononitrile unit can rotate. Consequently, coordination of the CN group with the surface rotates the conjugated 5,6‐difluoro‐3‐oxo‐2,3‐dihydro‐1*H*‐inden‐1‐ylidene moiety having a quasi‐planar geometry (Figures 2 a), lifting the F atoms and thus elongating the Pb‐F distances. However, the weak Pb‐F contacts (Figure S12) contribute to more intimate interactions between the HTM and the perovskite surface. iii) The lone pairs of electrons on sulfur atoms of the π‐bridges interact with the Pb^2+^ cations of the surface, which are similar to previously reported oligothiophene HTMs.^[23]^ The Pb‐S distances around 3.7 Å are below the sum of van der Waals radii of Pb and S, evidencing coordination interactions. iv) The average Pb‐O distances in the stabilized molecular conformation are about 3.0–3.5 Å, which is below or close to the sum of van‐der‐Waals radii of the interacting atoms. The average distances of the shortest contacts I‐X (X=O, N, S, F) are longer, being about 4 Å. In the face‐on configuration the hexyl units also tend to orient along the perovskite surface, which allows the site‐specific non‐covalent Pb‐X interactions to be maximized, since the corresponding parts of HTM can sufficiently approach the surface. Some hexyl units are unable to orient parallel to the surface due to the steric hindrance produced by other parts of CI‐TTIN‐2F (Figure 2 a), and sometimes the hexyl chains provide steric hindrance.

To elucidate the impact of CI‐TTIN‐2F on the electronic structure of the interface, a representative snapshot during the MD trajectory was selected to simulate the electronic structure using PBE0 density functional (Figure 2 b, c). The perovskite surface was represented by PbI_2_‐terminated slab, in which each Pb^2+^ ion can be considered as unsaturated, representing a defect. In the absence of CI‐TTIN‐2F on the perovskite surface, Pb‐centered trap‐like states were observed at the bottom of the conduction band of the perovskite (Figure 2 b). Interestingly, upon coverage of the perovskite with CI‐TTIN‐2F, these states disappear, with the contribution of the Pb^2+^ ions being “merged” with the continuous manifold of states at the bottom of the conduction band (Figure 2 c). Moreover, the empty CI‐TTIN‐2F‐centered states are situated below the conduction band manifold (Figure S13). Consequently, an electron from the bottom of the perovskite conduction band at the interface can migrate on the CI‐TTIN‐2F instead of recombining with the holes. Moreover, the participation of CI‐TTIN‐2F orbitals in the formation of the bottom of the conduction band favors electron delocalization over the perovskite/HTM interface, which in turn facilitates electron migration.

X‐ray photoelectron spectroscopy (XPS) was carried out on the CsPbI_3_ and CsPbI_3_/CI‐TTIN‐2F films to verify the calculations. High‐resolution Pb 4f spectra are shown in Figure 2 d. Apart from the two main Pb 4f_7/2_ and Pb 4f_5/2_ peaks, two additional peaks around 136.9 and 141.8 eV were observed in the CsPbI_3_ film attributed to the presence of the metallic Pb.^[24]^ The substantial metallic Pb species indicate the existence of iodide vacancies or under coordinated Pb^2+^ defects, which are likely to behave as non‐radiative carrier recombination centers and impede the solar cell performance. After depositing the CI‐TTIN‐2F HTM, the Pb 4f peaks shift to lower binding energies, and the two metallic Pb peaks are greatly restrained, demonstrating the strong interactions and the effective passivation of the CI‐TTIN‐2F HTM on the perovskite surface, in line with the MD and PDOS calculations. The influence of CI‐TTIN‐2F on the trap density of states of the CsPbI_3_ film was further investigated by space‐charge limited current (SCLC) characterization (Figure 2 e, f).^[25]^ The dark current density‐voltage curves show three representative regions consisting of a linear ohmic, a trap‐filled, and a trap‐free SCLC region. The trap‐filled limit voltage (*V*_TFL_) is reduced to 0.88 V from 1.27 V, and the corresponding trap density of states decrease to 3.04×10^15^ from 4.38×10^15^ cm^−3^ after depositing the CI‐TTIN‐2F HTM. These results confirm that CI‐TTIN‐2F can efficiently passivate the under coordinated Pb^2+^ defects on the CsPbI_3_ perovskite surface through the interactions identified by the calculations.

Since charge extraction properties are crucial for HTMs, we examined the charge‐carrier dynamics in pristine CsPbI_3_ and CsPbI_3_/HTM films using steady‐state photoluminescence (PL) and time‐resolved photoluminescence (TRPL) spectroscopy. The pristine CsPbI_3_ film exhibits the strongest PL emission centered around 725 nm (Figure S14). Significant quenching was observed in the presence of the HTM, and CI‐TTIN‐2F has the lowest PL intensity, suggesting rapid hole extraction across the interface and a superior hole extraction ability of CI‐TTIN‐2F compared to spiro‐OMeTAD. TRPL spectroscopy was used to delineate the carrier dynamics quantitatively (Figure 2 g). The pristine CsPbI_3_ film shows a longer lifetime (*τ*_1_=2.11 ns), which was reduced by the HTM due to the charge extraction. The CsPbI_3_/CI‐TTIN‐2F junction presents faster hole transfer (0.69 ns) than the CsPbI_3_/spiro‐OMeTAD interface (0.76 ns), most likely due to the deeper valence band maximum of CI‐TTIN‐2F and the stronger interfacial interactions between the Pb^2+^ ion on the perovskite surface and 2FIN units of CI‐TTIN‐2F compared to spiro‐OMeTAD.^[26]^ PL mapping images in Figure 2 h,i reveal a similar trend where the sample with CI‐TTIN‐2F displays lower integrated intensity than spiro‐OMeTAD. In addition, improved emission homogeneity was achieved by CI‐TTIN‐2F, which might result from the passivation of surface defects and the oriented face‐on stacking of the HTM.

**Figure 3:**
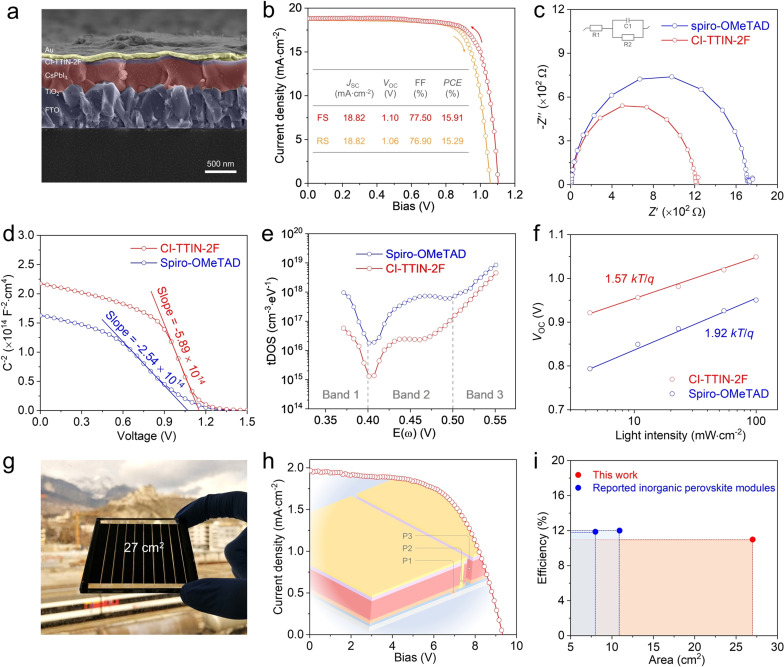
a) Cross‐section SEM image of the device with CI‐TTIN‐2F HTMs. b) The *J*–*V* curves of the CI‐TTIN‐2F‐based champion device. c) Nyquist plots, d) Mott‐Schottky plots, e) the trap density of states and f) *V*_OC_ as a function of light intensity of the CsPbI_3_ PSCs with different HTMs. g) Photograph of the CI‐TTIN‐2F‐based mini module. h) The *J*–*V* curve of the best‐performing module. The inset is the schematic illustration of the mini‐module device structure. i) PCEs of all‐inorganic perovskite modules versus module area from this work and previous reports.

Figure 3 a shows the cross‐section SEM image of the complete PSCs with an n‐i‐p structure comprising FTO/TiO_2_/CsPbI_3_/CI‐TTIN‐2F/Au, in which the thickness of the CI‐TTIN‐2F hole transport layer (HTL) was optimized at ≈50 nm. The detailed fabrication of the devices is described in the Supporting Information, and all the HTMs were employed without any dopant if not otherwise specified. Negligible change in grain sizes and morphologies of CsPbI_3_ films is observed after HTL deposition as shown in the surface‐section SEM images (Figure S15). The best‐performing CI‐TTIN‐2F‐based device shows a PCE of 15.91 % under reverse scan conditions, with a short‐circuit photocurrent density (*J*_SC_) of 18.82 mA cm^−2^, a *V*_OC_ of 1.10 V, and a fill factor (FF) of 77.50 %, and a PCE of 15.29 % in forward scan condition, with a *J*_SC_ of 18.82 mA cm^−2^, a *V*_OC_ of 1.06 V, and an FF of 76.90 % (Figure 3 b). The device performance is higher than that of the spiro‐OMeTAD‐based PSC with an efficiency of 11.44 % under reverse scan and 8.68 % in forward scan (Figure S16), reflecting the lower‐lying HOMO levels as well as the improved hole mobility and extraction for CI‐TTIN‐2F HTM. The two devices exhibit similar integrated photocurrent values (Figure S17), and the main difference in efficiency derives from the *V*_OC_ and FF. To explore the fundamental reasons, additional electrical characterization was performed on these devices. Electrochemical impedance spectroscopy (EIS) analysis reveals a smaller EIS resistance value for the CI‐TTIN‐2F‐based device relative to the spiro‐OMeTAD‐based device (Figure 3 c), demonstrating its better charge‐transfer behavior.^[19]^ The heterojunction properties at the perovskite/HTM interface were analyzed by capacitance‐voltage (*C*–*V*) measurements. The *C* ^−2^–*V* plots of the CI‐TTIN‐2F and spiro‐OMeTAD‐based device are depicted in Figure 3 d, following the Mott‐Schottky Equation 1:

(1) $$\frac{1}{C^{2}}=\frac{2}{e\epsilon \epsilon_{0}N_{D}}\left(V_{bi}-V-\frac{k_{B}T}{e}\right)$$

where *C* is the capacitance, *V*_bi_ refers to the built‐in potential, *N*_D_ represents the charge density, and *V* stands for the applied voltage. *k*_B_, *T*, *e*, *ϵ*, and *ϵ*_0_ are the Boltzmann constant, thermodynamic temperature, elementary charge, relative dielectric constant and vacuum permittivity, respectively. The *V*_bi_ caused by the carrier diffusion is crucial for charge injection in solar cells, which is estimated to be 1.15 V for the CI‐TTIN‐2F‐based device, outperforming the spiro‐OMeTAD‐based device (1.07 V), consistent with the decreased energy level of CI‐TTIN‐2F (Figure 1 b). Therefore, CI‐TTIN‐2F extends the depleted region and enhances the driving force for carrier injection, which directly contributes to the increased *V*_OC_.^[27]^ Moreover, the larger slope of the Mott‐Schottky plot for the CI‐TTIN‐2F‐based device suggests a lower interfacial charge density (3.79×10^16^ cm^−3^ vs. 8.79×10^16^ cm^−3^) and thus improved charge extraction. The higher charge accumulation at the perovskite/spiro‐OMeTAD interface also explains the larger hysteresis.^[28]^ The trap density of states (*t*DOS) was also deduced from the angular frequency‐dependent capacitance for the fabricated devices (Figure 3 e). The CI‐TTIN‐2F‐based device demonstrates reduced trap states compared with the spiro‐OMeTAD‐based device over the whole defect energy region. This reduction derives from the effective surface passivation of CI‐TTIN‐2F, which is confirmed by the removal of trap states (Figure 2 b). Accordingly, trap‐assisted recombination is efficiently suppressed, as validated by the smaller ideality factor of 1.57 for the CI‐TTIN‐2F‐based device compared with 1.92 for the spiro‐OMeTAD device (Figure 3 f). Hence, CI‐TTIN‐2F gives rise to advantageously accelerated interface charge transfer, diminishes surface defects, and suppresses trap‐assisted recombination, which reduces energy loss and thus enhances device performance, especially for *V*_OC_ and FF.

To demonstrate large‐scale application of the CI‐TTIN‐2F HTM, inorganic CsPbI_3_ perovskite modules with an active area of 27 cm^2^ were fabricated (Figure 3 g). The modules were patterned by the P1, P2, and P3 laser‐scribing method (Figure 3 h), consisting of nine sub‐cells. Notably, the CsPbI_3_ PSC module achieves a PCE of 10.98 % employing the CI‐TTIN‐2F HTM, with a *J*_SC_ of 1.96 mA cm^−2^, a *V*_OC_ of 9.36 V, and an FF of 60 %. The similar *V*_OC_ of each sub‐cell with a single cell implies the good uniformity of the HTM layer over a large area. The reduced *J*_SC_ and FF can be attributed to the greater series resistance because of the longer carrier transport paths and the device nonuniformities. Moreover, the perovskite solar module with the CI‐TTIN‐2F HTM shows a steady output PCE of 10.80 % under the AM 1.5 G illumination for 250 s, which is consistent with that calculated from *J*–*V* scanning (Figure S18). The performance of the reported inorganic perovskite modules versus device area are summarized in Figure 3 i.^[29]^ The efficiency of the CI‐TTIN‐2F‐based module is comparable with that of previous reports while showing the largest module area.

**Figure 4:**
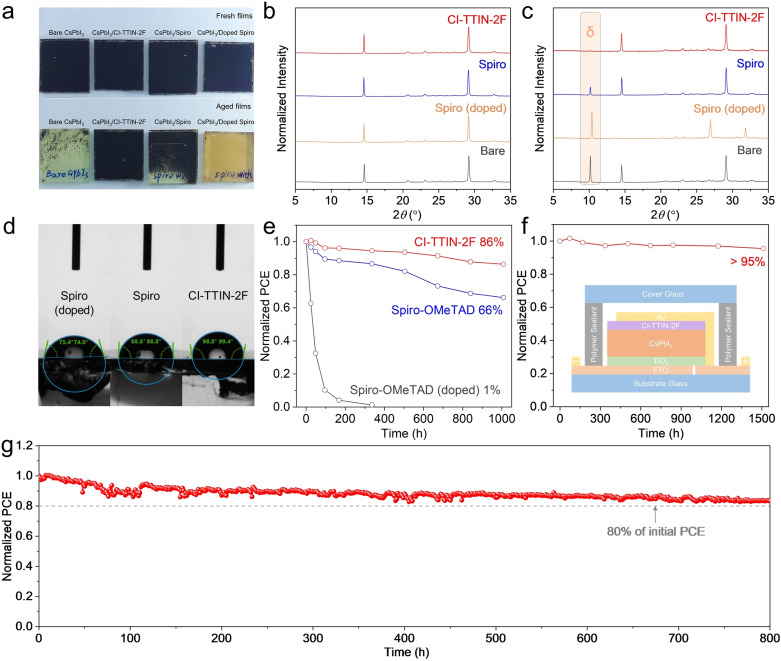
a) Photos of the CsPbI_3_ films with and without different HTMs before and after exposure to RH of ≈50 % for 480 min. XRD pattern of the CsPbI_3_ films with and without different HTMs (b) before and (c) after exposure to RH of ≈20 % for 360 h. d) Water contact angles of different HTLs. e) Stability under ambient (≈20 % RH) for the unencapsulated CsPbI_3_ PSCs with different HTMs. f) Stability of encapsulated CI‐TTIN‐2F‐based device in air (≈50 % RH), the inset is the illustration of the cross sections of the respective encapsulation schemes. g) Operational stability of the CsPbI_3_ device with CI‐TTIN‐2F HTMs under a constant one sun illumination at the maximum power point without a UV filter in nitrogen atmosphere.

Moisture‐induced phase transitions of the perovskite film are a critical degradation path for all‐inorganic PSCs. To examine the effects of HTL incorporation on the phase stability of perovskite films, we tracked the absorbance evolution of CsPbI_3_ films under a controlled relative humidity (RH) of ≈50 %, and photographs of the perovskite films stored for different times are shown in Figure 4 a and Figure S19. Remarkably, the CsPbI_3_ film covered with doped spiro‐OMeTAD is bleached within only 20 min, which is even more rapid than that of the bare CsPbI_3_ film (100 min). The degradation onset is significantly retarded to 180 min when the hydrophilic dopants were removed from the spiro‐OMeTAD HTL composition. Specially, no obvious change in color is observed from the CsPbI_3_ film with the CI‐TTIN‐2F HTM after aging for 480 min. In addition, the long‐term phase stability of the films under ambient conditions with an RH of ≈20 % was further investigated by X‐ray diffraction (XRD). All the fresh CsPbI_3_ films show a typical black phase with two main specific peaks located at 14.6° and 29.2° which are assigned to (110) and (220) planes, respectively (Figure 4 b). The CsPbI_3_ films with various HTLs behave differently after aging for 360 h (Figure 4 c). A characteristic peak assigned to the δ phase at 10.2° was observed in the bare CsPbI_3_ film after aging.^[30]^ Significantly, the upper spiro‐OMeTAD HTL with and without dopants accelerate and decelerate, respectively, the phase transition of the CsPbI_3_ perovskite structure to the yellow δ phase. Thus, these hygroscopic dopants not only eliminate the advantage of hydrophobicity of the upper spiro‐OMeTAD layer but also further accelerate the phase transition of the CsPbI_3_ films. When coated with the dopant‐free CI‐TTIN‐2F HTL, the CsPbI_3_ film remains in the black phase and is almost identical to the fresh film, indicating that CI‐TTIN‐2F is capable of protecting the metastable perovskite films from moisture penetration. To probe surface property of the HTLs, contact angles of water droplets on the CsPbI_3_/HTLs samples were measured (Figure 4 d). The CI‐TTIN‐2F HTL showed a much higher contact angle of ≈99° than those of spiro‐OMeTAD with (≈75°) and without dopants (≈88°), and the improved hydrophobicity can be in part attributed to the introduced fluorinated 1,1‐dicyanomethylene‐3‐indanone acceptor units in the D‐π‐A structure.

The stability of unencapsulated CsPbI_3_ PSCs employing different HTMs was monitored in an ambient atmosphere with an RH of ≈20 % for 1000 hours. Figure 4 e presents the normalized PCEs of the devices as a function of storage time, and the device performances were periodically measured in ambient air. For comparison, we also fabricated CsPbI_3_ devices using spiro‐OMeTAD with conventional dopants and the best‐performing device exhibited a PCE of 17.77 % with a *J*_SC_ of 19.53 mA cm^−2^, a *V*_OC_ of 1.12 V, and an FF of 78.70 % (Figure S20). However, the ambient stability monitored under ambient conditions reveals that the doped spiro‐OMeTAD‐based device suffers a sharp drop up to <10 % of the initial PCE within only 100 h due to the moisture‐sensitive dopants. Furthermore, the dopant‐free spiro‐OMeTAD‐based device retains 66 % of the pristine performance after 1000 hours. Encouragingly, the CI‐TTIN‐2F‐based device maintains 86 % of its initial PCE after 1000 h exposure. To further enhance device stability, we encapsulated the CI‐TTIN‐2F‐based device and a cross‐section of the encapsulation Scheme is illustrated in the inset in Figure 4 f. Successfully, the device displays improved stability, and the efficiency drops by only 5 % after storing under a controlled ≈50 % RH for over 1500 h. Moreover, we examined the operational stability of the unencapsulated CI‐TTIN‐2F‐based device under a constant one sun illumination (AM1.5G) at 25 °C in a nitrogen atmosphere at the maximum powerpoint (Figure 4 g). The CsPbI_3_ PSC with CI‐TTIN‐2F maintains over 80 % of the initial PCE under light soaking for 800 h, demonstrating a superior photochemical stability based on this new CI‐TTIN‐2F HTM.

## Conclusion

We synthesized a new dopant‐free HTM named CI‐TTIN‐2F with a D‐π‐A molecular configuration. The suitable optoelectronic properties and energy‐level alignment endow CI‐TTIN‐2F with excellent charge collection properties. In addition, joint experimental and theoretical studies suggest that CI‐TTIN‐2F has the capacity for multisite passivation effects on defective CsPbI_3_ surfaces due to the presence of various heteroatoms. As a result, all‐inorganic CsPbI_3_ PSCs with dopant‐free CI‐TTIN‐2F HTM demonstrate a high PCE of 15.9 %, along with 86 % efficiency retention after 1000 hours under ambient conditions. These results indicate an excellent compatibility of CI‐TTIN‐2F with all‐inorganic PSCs. Notably, the largest all‐inorganic perovskite solar module was fabricated using the CI‐TTIN‐2F HTM, and exhibited an efficiency of 11.0 % with an area of 27 cm^2^. This study provides a new design strategy toward efficient dopant‐free HTMs with multisite passivation effects that stabilize all‐inorganic PSCs to facilitate future scale‐up.

## Conflict of interest

The authors declare no conflict of interest.

## Supporting information

As a service to our authors and readers, this journal provides supporting information supplied by the authors. Such materials are peer reviewed and may be re‐organized for online delivery, but are not copy‐edited or typeset. Technical support issues arising from supporting information (other than missing files) should be addressed to the authors.

Supporting Information
