# Supplementary material for: Dopant‐Free Hole Transport Materials Afford Efficient and Stable Inorganic Perovskite Solar Cells and Modules
Source: Angew Chem Int Ed Engl. 2021 Aug 7;60(37):20489–97. doi: 10.1002/anie.202107774 (PMC8456866; doi:10.1002/anie.202107774)
Supplement: Supplementary file 1 — Supporting Information [file ANIE-60-20489-s001.pdf]

Angewandte Chemie
Eine Zeitschrift der Gesellschaft Deutscher Chemiker

Supporting Information

Dopant-Free Hole Transport Materials Afford Efficient and Stable Inorganic Perovskite Solar Cells and Modules

Cheng Liu+, Cansu Igci+, Yi Yang+, Olga A. Syzgantseva, Maria A. Syzgantseva, Kasparas Rakstys, Hiroyuki Kanda, Naoyuki Shibayama, Bin Ding, Xianfu Zhang, Vygintas Jankauskas, Yong Ding, Songyuan Dai,* Paul J. Dyson,* and Mohammad Khaja Nazeeruddin**

anie_202107774_sm_miscellaneous_information.pdf
anie_202107774_sm_Movie_SI.mp4

Supporting Information

Experimental Procedures

Materials:
The CsI, PbI_2, and hydroiodic acid were purchased from Alfa Aesar. 2,2′,7,7′-tetrakis(N,N-di-p-methoxyphenyl-amino)-9,9′-spirobifluorene (spiro-OMeTAD), lithium bis(trifluoromethane)sulfonamide (LiTFSI), tris(2-(1H-pyrazol-1-yl)-4-tert-butyl pyridine) cobalt(III) tris(bis(trifluoromethyl sulfonyl)imide) (FK209) and 4-tert-butyl pyridine (TBP) were purchased from Xi'an Polymer Light Technology Corp. N,N-dimethylformamide (DMF, 99.9%) and chlorobenzene (99.8%) were from Sigma-Aldrich. The $DMAPbI_3$ was prepared by mixing PbI_2 and 57% w/w hydroiodic acid (molar ratio 1:1.5) in DMF with stirring for 2 h at 60°C. The as-achieved solution was rotary evaporated at 75 °C to remove the excess solutions. Finally, the resulting solid was washed with diethyl ether and the collected powders are dried in a vacuum oven overnight.
Device Fabrication:
The TiO_2 compact layer was firstly deposited on clean FTO substrates by spray pyrolysis from a precursor solution of diluted titanium diisopropoxide bis(acetylacetonate) (TAA) in isopropanol at 450 °C. The 0.6 M $CsPbI_3$ precursor solution was prepared by dissolving stoichiometric CsI and $DMAPbI_3$ with a 1:1 molar ratio in DMF. The precursor solution was spun onto the room-temperature (RT) substrates at 1000 rpm for 10 s with a ramping rate of 500 rpm·s^{-1} and subsequently at 2500 rpm for 30 s with a ramping rate of 1000 rpm·s^{-1}. After spin-coated, the substrates were transferred onto a hotplate with 180°C for 25 min to obtain uniform $CsPbI_3$ films. Dopant-free hole transport materials (HTMs) with the concentration of 15 mg/mL (Cl-TTIN-2F in tetrachloroethane or spiro-OMeTAD in chlorobenzene) were subsequently spin-coated at 2000 rpm for 20 s. For comparison, a conventional spiro-OMeTAD with dopants which prepared by dissolving 75 mg spiro-OMeTAD in chlorobenzene solution with 32 μL TBP, 20 μL Li-TFSI/acetonitrile (1.8 M), and 8 μL FK209/acetonitrile (1 M), was spun to the perovskite substrates at 3500 rpm for 20 s. Finally, the devices were finished by the evaporation of 70 nm Au electrode. For module fabrication, 6.5 cm × 7 cm FTO substrates were patterned by laser with nine sub-cells connected in series. The P1 lines were patterned on FTO substrates first with a width of 50 μm. The film deposition processes were the same with the small solar cells. Then the laser scribing was performed twice before (P2, 500 μm) and after (P3, 200 μm) gold evaporation to complete the module fabrication.
Device characterization:
The *J-V* characteristics of the photovoltaic devices were measured in ambient air with RH ~30% under an AM 1.5 simulated light source connected to a source meter (Keithley 2400), calibrated by an NREL- certified KG5 filtered Si reference diode. A black metal mask defined the device area as 0.16 cm^2. EQE was characterized by an Enli Technology EQE measurement system with a dual xenon/quartz halogen light source. Steady-state PL and TRPL were performed by Edinburgh FLS-980 fluorescence spectrometer (Edinburgh Instruments, UK). Confocal PL intensity maps were recorded with a laser confocal Raman spectrometer (Princeton Instruments, Acton Standard Series SP-2558) and a 485 nm laser (PicoQuant LDH-P-C-485, 0.4 mW with a 1% optical density filter) using a home-built confocal microscope. The EIS was performed with an SP-200 potentiostat (BioLogic) in the dark condition with bias of 0.8 V. The thermal admittance spectroscopy was conducted at a frequency range of 1 Hz to 1 MHz at a temperature of 300 K. The GIWAXS was measured through a Huber diffractometer and photon energy of 12.39 keV (1.0 Å) at a fixed incident angle on the order of 0.12° through a Huber diffractometer by using synchrotron radiation at beamline BL19B2 of SPring-8 with PILATUS 300K 2D X-ray detectors. Cyclic voltammetry (CV) was measured with a Biologie SP-200 cyclic voltammeter with a standard three-electrode configuration. Thermogravimetric analysis (TGA) data were collected using TGA 4000 from PerkinElmer. MS were recorded on 6530 Accurate-Mass Q-TOF LC/MS (Agilent Technologies) using electrospray ionization (ESI) and atmospheric pressure photoionization (APPI) techniques or Axima-CFR plus (Shimadzu) using matrix-assisted laser desorption/ionization (MALDI) technique.
Computational details:
All simulations are performed within the density functional theory using CP2K code[5], employing localized atomic DZVP-MOLOPT basis sets[6], auxiliary plane wave basis set with 600 Ry cutoff for interfaces, and 450 Ry for isolated molecules and Goedecker-Teter-Hutter pseudopotentials[7] for the description of the core region. The interaction of the HTM was modeled with PbI_2-terminated perovskite surface, obtained from the bulk structure of the tetragonal β-$CsPbI_3$ phase having the space group P4/mbm, as reported by Marronnier *et al*.[8] For this purpose, Born-Oppenheimer molecular dynamics simulations of the Cl-TTIN-2F molecule deposited on top of perovskite slab were performed employing the Perdew-Burke-Ernzerhof (PBE)[1] density functional with Grimme's dispersion correction[2]. The molecule was allowed to freely relax on top of the perovskite surface at the constant temperature of 300 K, maintained by the velocity rescaling algorithm. PBE-D3[1, 2] , B3LYP[9-11], HSE06[12,] and PBE0[3, 4] density functionals were benchmarked to describe the electronic

structure of isolated HTM molecules. Auxiliary density matrix method (ADMM)[13] was used for the calculations with the hybrid or range-separated functionals. The structures were visualized with VESTA[14] and VMD[15] programs.

The general synthetic procedure of CI-TTIN-2F:

Synthesis of the intermediate 5'',5'''',5''''''-(5,10,15-trihexyl-10,15-dihydro-5H-diindolo[3,2-a:3',2'-c]carbazole-3,8,13-triyl)tris(3,3''-dihexyl-[2,2':5',2''-terthiophene]-5-carbaldehyde) compounds 4 was accomplished starting from commercially available and inexpensive precursor 2-indolinone according to the reported method with a small modification.[16] The designed acceptor unit was synthesized from commercially available 5,6-Difluoro-2-benzofuran-1,3-dione with following the reported method.[17] It reduced in the presence of tert-butyl acetoacetate and triethyl amine to difluorinated indanedione then dicyanovinylene moiety was incorporated in one carbonyl to get 2-(5,6-difluoro-3-oxo-2,3-dihydro-1H-inden-1-ylidene)malononitrile. Then, compound 4 (0.25 g, 0.13 mmol, 1eq.) and 2-(5,6-difluoro-3-oxo-2,3-dihydro-1*H*-inden-1-ylidene)malononitrile (0.29 g, 1.30 mmol, 10 eq.) were dissolved in chloroform (30 mL) and a few drops of pyridine base were added. The reaction mixture was stirred overnight at reflux temperature. The reaction was quenched with water and was acidified with few drops of conc. HCl. The organic layer was extracted with chloroform, dried over anhydrous magnesium sulfate, filtered and concentrated under reduced pressure. The resulting residue was purified by column chromatography on silica gel (2% ethanol in chloroform) to yield dark green solid. 2,2',2''-((2Z,2'Z,2''Z)-(((5,10,15-trihexyl-10,15-dihydro-5H-diindolo[3,2-a:3',2'-c]carbazole-3,8,13-triyl)tris(3,3''-dihexyl-[2,2':5',2''-terthiophene]-5'',5-diyl))tris(methaneylylidene))tris(5,6-difluoro-3-oxo-2,3-dihydro-1*H*-indene-2,1-diylidene))trimalononitrile CI-TTIN-2F was obtained with a good yield (0.18 g, 53%). Due to intermolecular aggregation and strong π- π interaction, ^{1}H and ^{13}C NMR spectra could not be obtained. $C_{153}H_{147}F_6N_9O_3S_9$ [M$^+$] Exact Mass =2559.90, MS (MALDI-TOF) =2560.274 Elemental analysis calcd (%) for $C_{153}H_{147}F_6N_9O_3S_9$: C, 71.72; H, 5.78; N, 4.92; S, 11.26. Found: C, 70.86; H, 5.28; N, 4.73; S, 10.51.

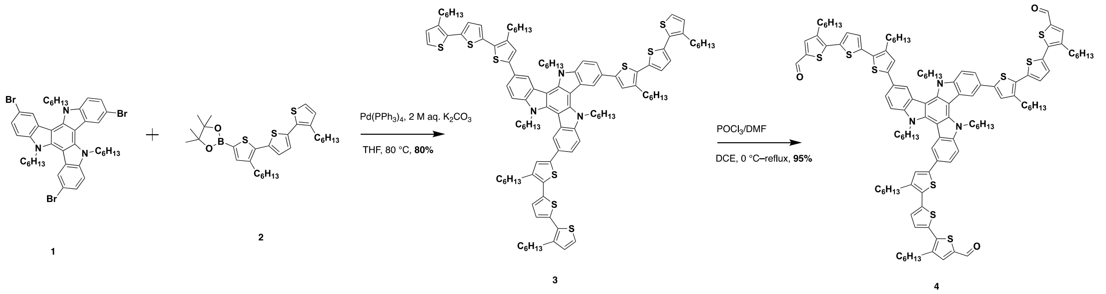

Figure S1. Synthetic route of 5'',5'''',5''''''-(5,10,15-trihexyl-10,15-dihydro-5H-diindolo[3,2-a:3',2'-c]carbazole-3,8,13-triyl)tris(3,3''-dihexyl-[2,2':5',2''-terthiophene]-5-carbaldehyde).

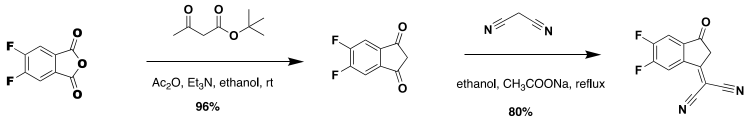

Figure S2. Synthetic route of 2-(5,6-difluoro-3-oxo-2,3-dihydro-1*H*-inden-1-ylidene)malononitrile.

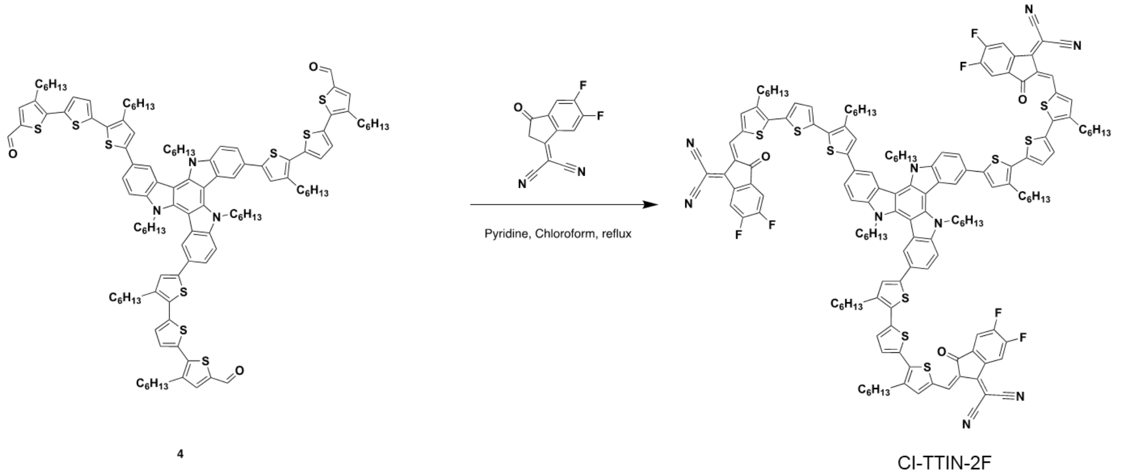

Figure S3. Synthetic routes of CI-TTIN-2F.

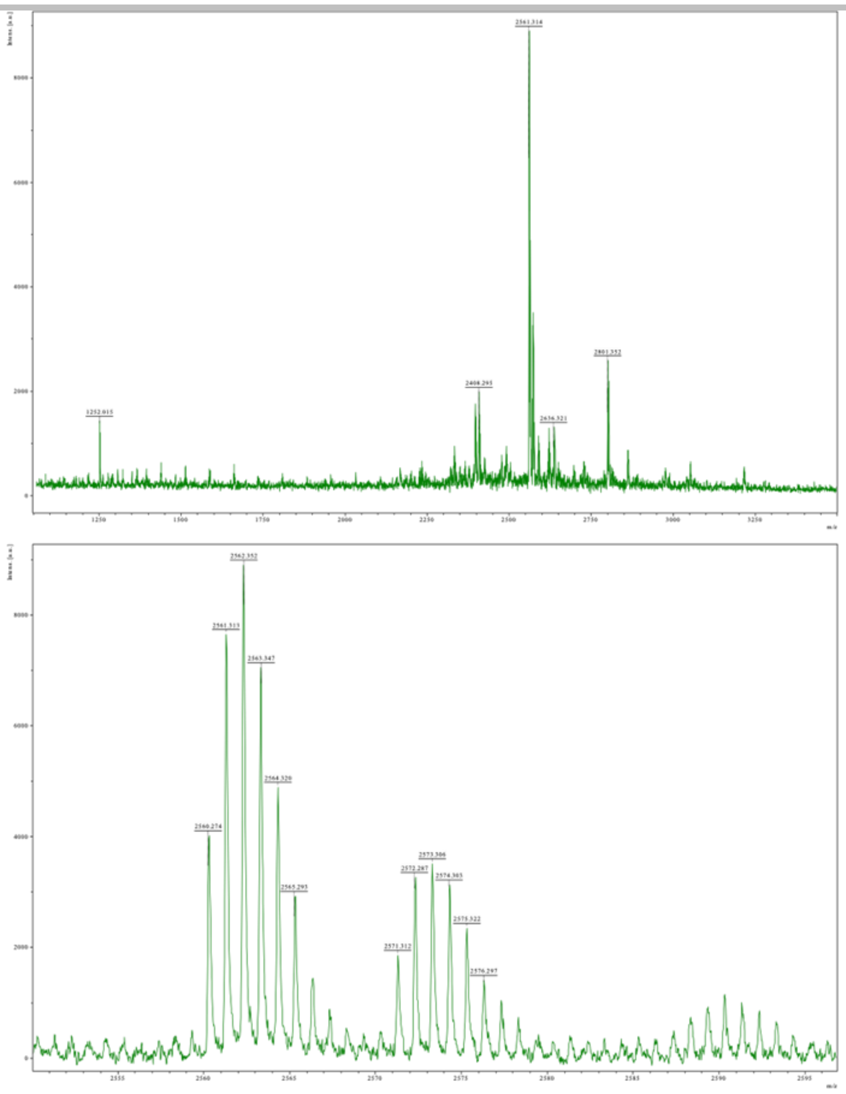

Figure S4. MALDI-TOF-MS spectra in wide and narrow mass ranges of CI-TTIN-2F.

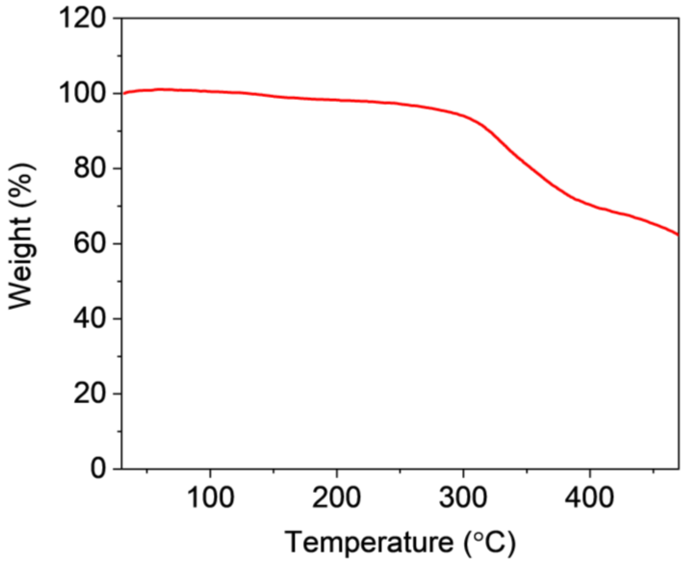

Figure S5. TGA curves of CI-TTIN-2F under nitrogen atmosphere with a heating rate of 20°C/min.

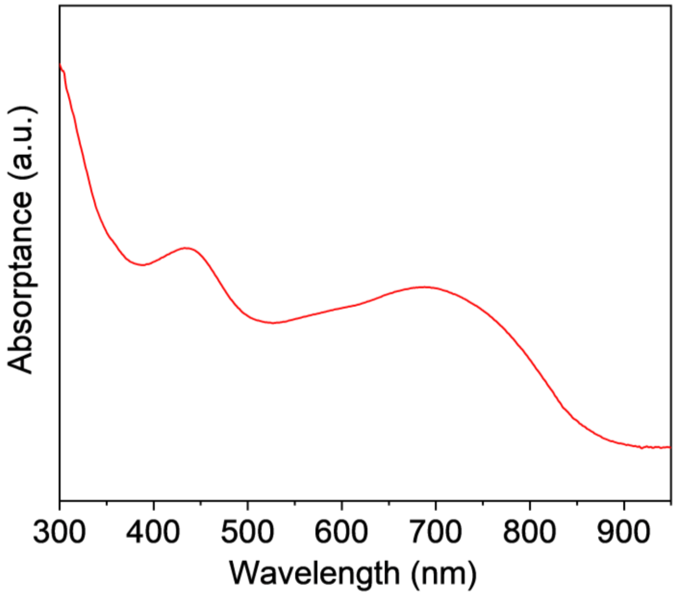

Figure S6. UV-Vis absorption spectra of CI-TTIN-2F thin films.

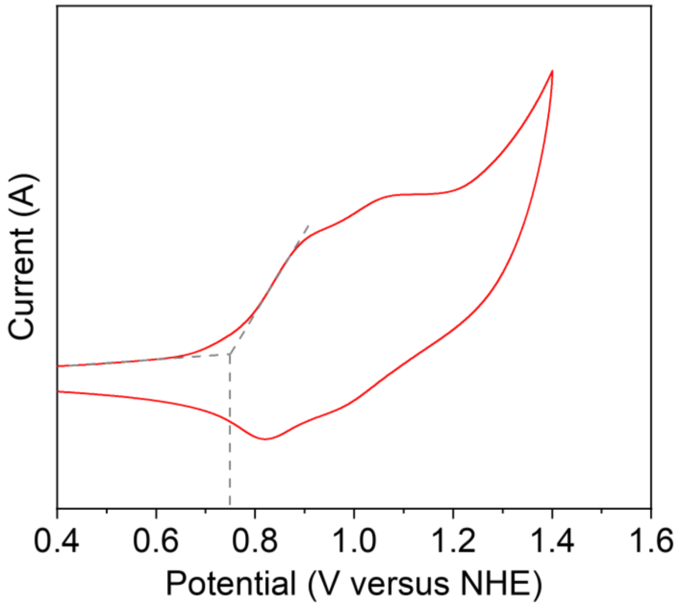

Figure S7. Cyclic voltammograms of the CI-TTIN-2F HTMs measured in 0.1 M n-Bu4NPF6 solution in tetrahydrofuran.

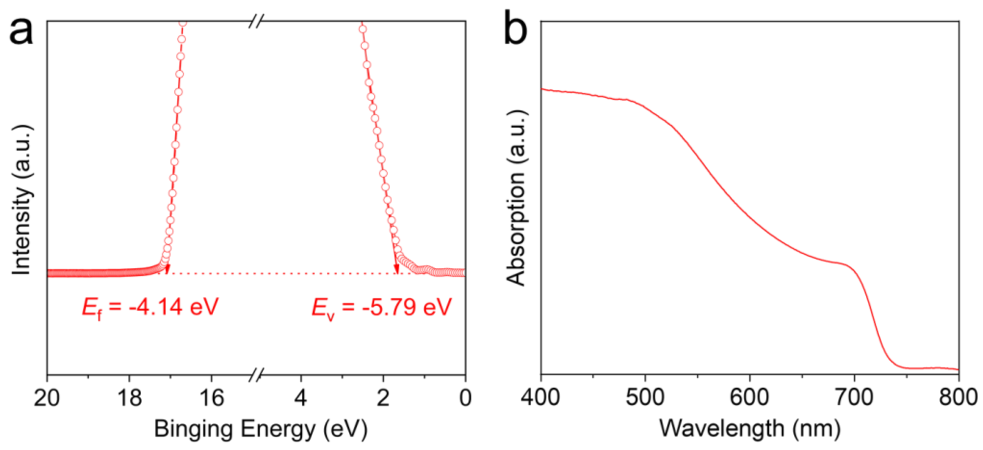

Figure S8. (a) UPS and (b) UV-vis spectra of the $CsPbI_3$ films.

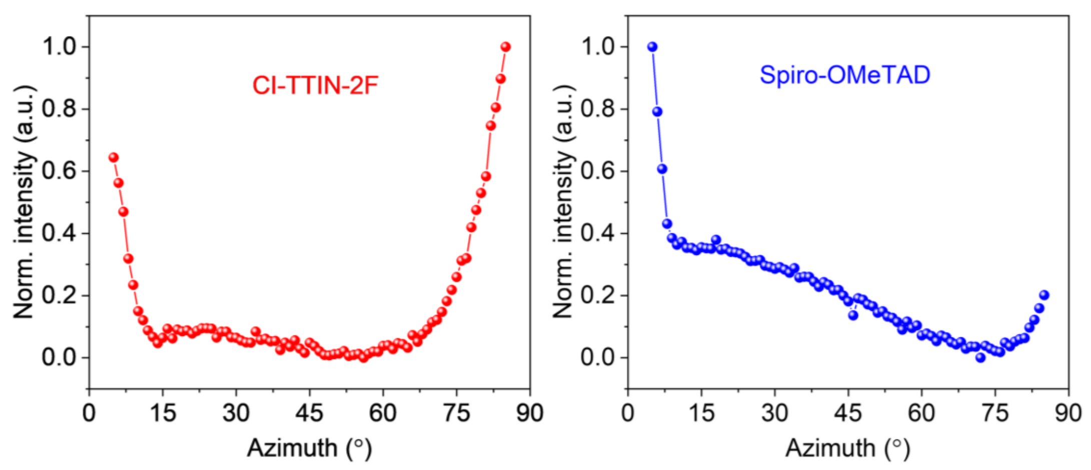

Figure S9. The pole plots of the azimuth angle integrated around q_z = 3.5 Å^{-1} of Cl-TTIN-2F and spiro-OMeTAD HTMs.

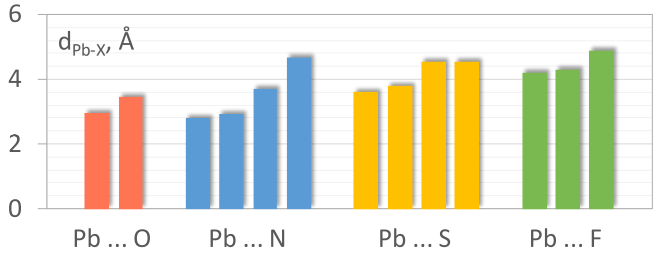

Figure S10. Average distances Pb-X, where X = O, N, S, F of the HTM deposited on perovskite surface.

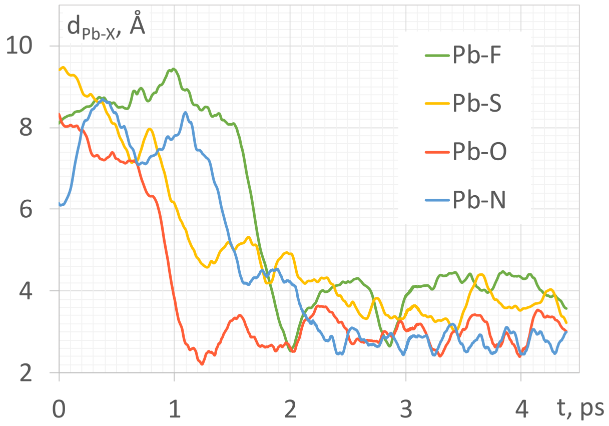

Figure S11. Evolution of distances Pb-X (X = O, N, S, F) during the process of Cl-TTIN-2F deposition on top of the perovskite surface: comparison between different contacts.

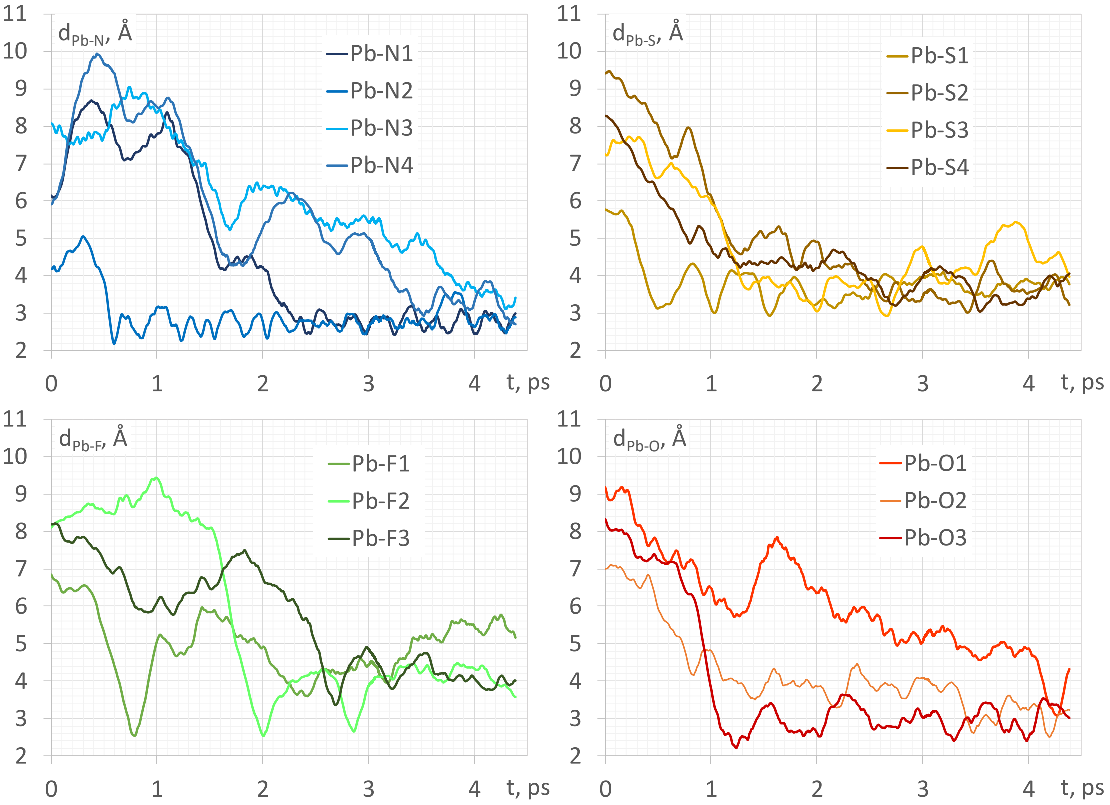

Figure S12. Evolution of distances Pb-X (X = O, N, S, F) during the process of Cl-TTIN-2F deposition on top of the perovskite surface: several contacts of the same type Pb-X*i*.

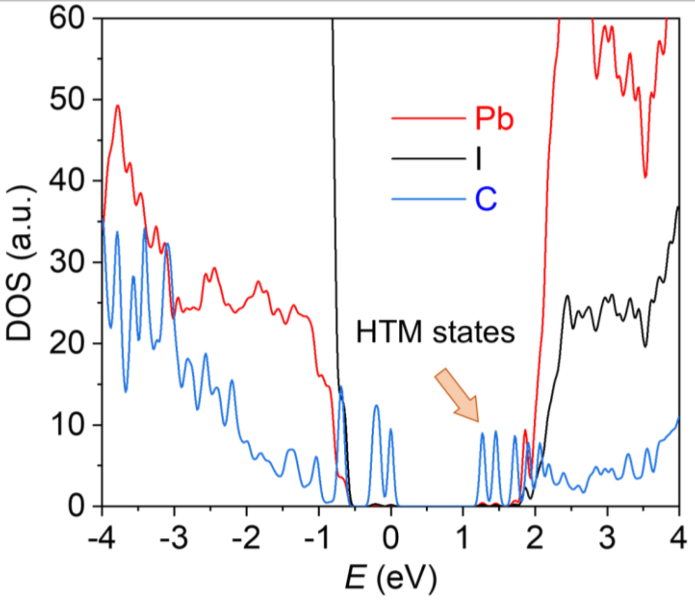

Figure S13. Electronic structure of the Cl-TTIN-2F/perovskite interface, calculated using PBE0 density functional.

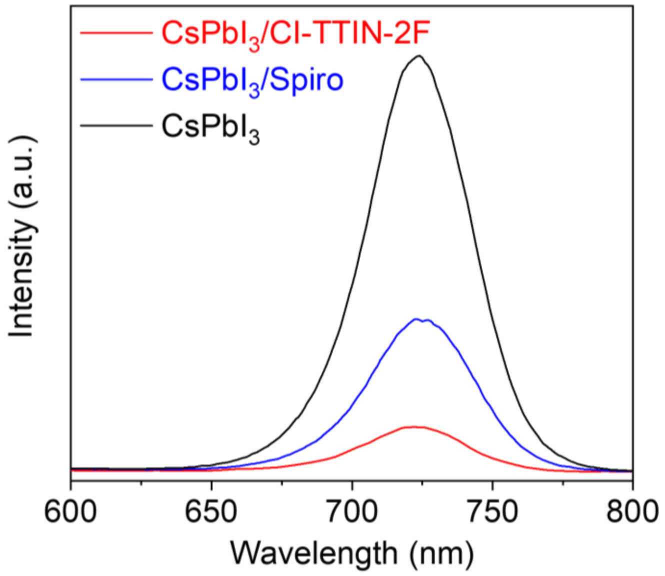

Figure S14. Steady-state photoluminescence (PL) spectra of $CsPbI_3$ films with and without different HTMs.

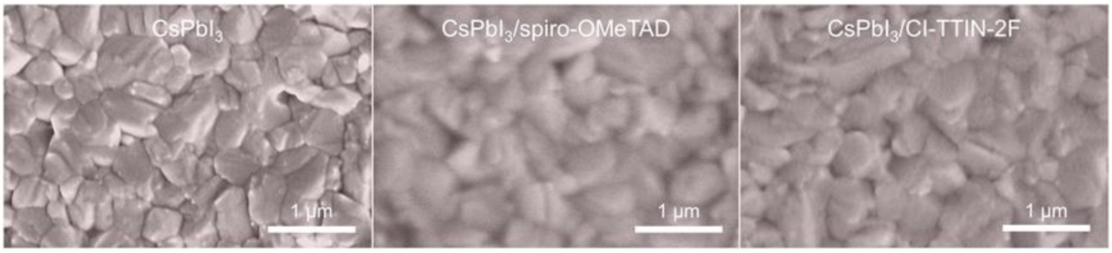

Figure S15. Surface-section SEM images of $CsPbI_3$ films with and without different HTMs.

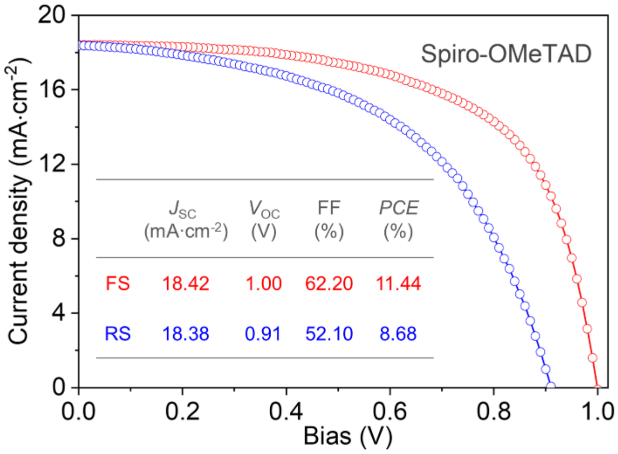

Figure S16. *J-V* curves of $CsPbI_3$ PSCs with dopant-free spiro-OMeTAD HTMs.

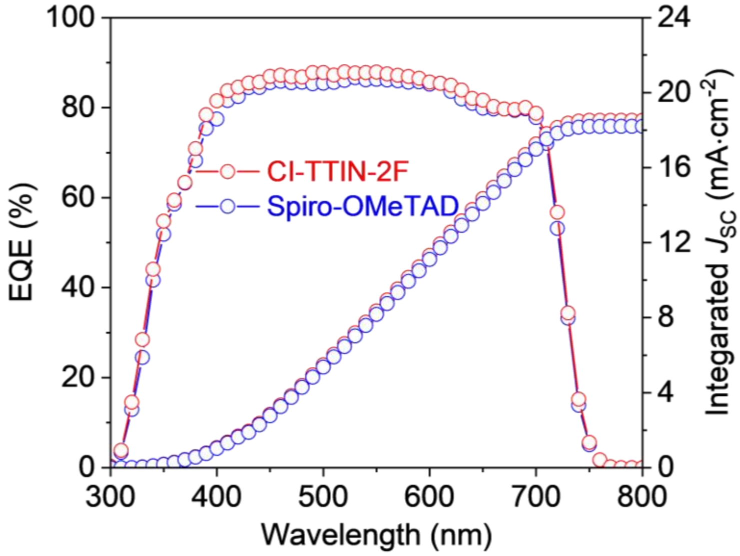

Figure S17. EQE of perovskite solar cells with various HTMs.

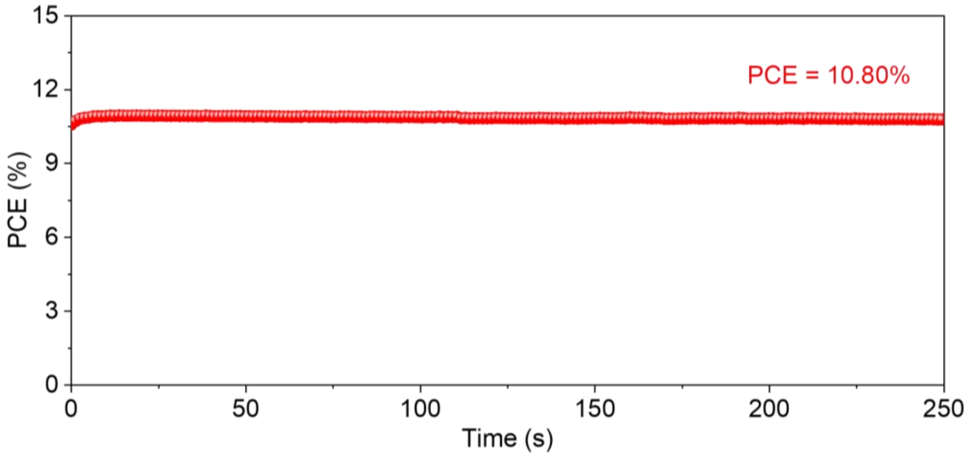

Figure S18. PCE output at a maximum power point of 6 V for the CI-TTIN-2F-based perovskite solar module.

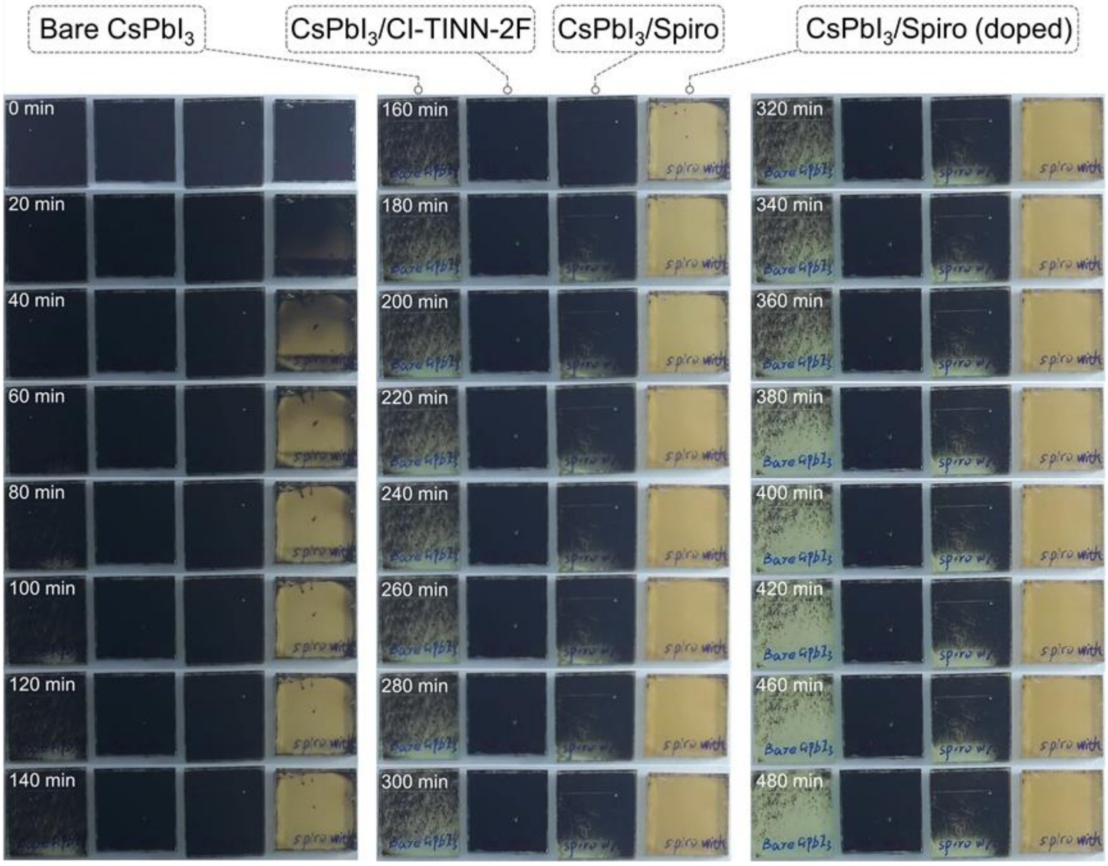

Figure S19. Photos of the $CsPbI_3$ films with and without different HTMs exposed to relative humidity (RH) of ~50% for a different time.

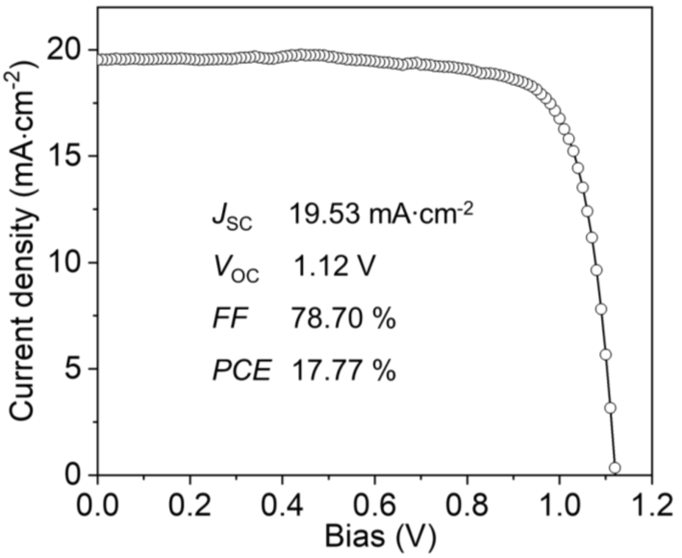

Figure S20. *J-V* curves of $CsPbI_3$ PSC with doped spiro-OMeTAD HTMs.

Table 1. Energies of HOMO and LUMO in eV.

	HOMO	LUMO
PBE-D3	-4.60	-3.58
PBE0@PBE-D3	-5.23	-3.29
PBE0	-5.23	-3.22

Table 2. Energy levels (in eV) of CI-TTIN-2F, computed with different density functionals on top of geometry optimized with PBE-D3 functional.

	HOMO	LUMO
PBE-D3	-4.60	-3.58
PBE0@PBE-D3	-5.23	-3.29
B3LYP@PBE-D3	-5.11	-3.23
HSE06@PBE-D3	-4.97	-3.53

References

[1] Perdew, J. P.; Burke, K.; Ernzerhof, M., Generalized gradient approximation made simple. *Physical Review Letters* **1996,** *77* (18), 3865-3868.

[2] Grimme, S.; Antony, J.; Ehrlich, S.; Krieg, H., A consistent and accurate ab initio parametrization of density functional dispersion correction (DFT-D) for the 94 elements H-Pu. *Journal of Chemical Physics* **2010,** *132* (15), 154104.

[3] Perdew, J. P.; Ernzerhof, M.; Burke, K., Rationale for mixing exact exchange with density functional approximations. *Journal of Chemical Physics* **1996,** *105* (22), 9982-9985.

[4] Adamo, C.; Barone, V., Toward reliable density functional methods without adjustable parameters: The PBE0 model. *Journal of Chemical Physics* **1999,** *110* (13), 6158-6170.

[5] Kühne, T. D.; Iannuzzi, M.; Del Ben, M.; Rybkin, V. V.; Seewald, P.; Stein, F.; Laino, T.; Khaliullin, R. Z.; Schütt, O.; Schiffmann, F.; Golze, D.; Wilhelm, J.; Chulkov, S.; Bani-Hashemian, M. H.; Weber, V.; Borštnik, U.; Taillefumier, M.; Jakobovits, A. S.; Lazzaro, A.; Pabst, H.; Müller, T.; Schade, R.; Guidon, M.; Andermatt, S.; Holmberg, N.; Schenter, G. K.; Hehn, A.; Bussy, A.; Belleflamme, F.; Tabacchi, G.; Glöß, A.; Lass, M.; Bethune, I.; Mundy, C. J.; Plessl, C.; Watkins, M.; VandeVondele, J.; Krack, M.; Hutter, J., CP2K: An electronic structure and molecular dynamics software package - Quickstep: Efficient and accurate electronic structure calculations. *The Journal of Chemical Physics* **2020,** *152* (19), 194103.

[6] VandeVondele, J.; Hutter, J., Gaussian basis sets for accurate calculations on molecular systems in gas and condensed phases. *Journal of Chemical Physics* **2007,** *127* (11), 114105.

[7] Goedecker, S.; Teter, M.; Hutter, J., Separable dual-space Gaussian pseudopotentials. *Physical Review B* **1996,** *54* (3), 1703-1710.

[8] Marronnier, A.; Roma, G.; Boyer-Richard, S.; Pedesseau, L.; Jancu, J. M.; Bonnassieux, Y.; Katan, C.; Stoumpos, C. C.; Kanatzidis, M. G.; Even, J., Anharmonicity and Disorder in the Black Phases of Cesium Lead Iodide Used for Stable Inorganic Perovskite Solar Cells. *Acs Nano* **2018,** *12* (4), 3477-3486.

[9] Becke, A. D., Density-functional exchange-energy approximation with correct asymptotic-behavior. *Physical Review A* **1988,** *38* (6), 3098-3100.

[10] Lee, C. T.; Yang, W. T.; Parr, R. G., Development of the colle-salvetti correlation-energy formula into a functional of the electron-density. *Physical Review B* **1988,** *37* (2), 785-789.

[11] Vosko, S. H.; Wilk, L.; Nusair, M., Accurate spin-dependent electron liquid correlation energies for local spin-density calculations - a critical analysis. *Canadian Journal of Physics* **1980,** *58* (8), 1200-1211.

[12] Krukau, A. V.; Vydrov, O. A.; Izmaylov, A. F.; Scuseria, G. E., Influence of the exchange screening parameter on the performance of screened hybrid functionals. *Journal of Chemical Physics* **2006,** *125* (22), 224106.

[13] Guidon, M.; Hutter, J.; VandeVondele, J., Auxiliary Density Matrix Methods for Hartree-Fock Exchange Calculations. *Journal of Chemical Theory and Computation* **2010,** *6* (8), 2348-2364.

[14] Momma, K.; Izumi, F., VESTA 3 for three-dimensional visualization of crystal, volumetric, and morphology data. *Journal of Applied Crystallography* **2011,** *44*, 1272-1276.

[15] Humphrey, W.; Dalke, A.; Schulten, K., VMD: Visual molecular dynamics. *Journal of Molecular Graphics* **1996,** *14* (1), 33-38.

[16] Rakstys, K.; Paek, S.; Gao, P.; Gratia, P.; Marszalek, T.; Grancini, G.; Cho, K. T.; Genevicius, K.; Jankauskas, V.; Pisula, W.; Nazeeruddin, M. K., Molecular engineering of face-on oriented dopant-free hole transporting material for perovskite solar cells with 19% PCE. J*ournal of Materials Chemistry A* **2017**, *5* (17), 7811-7815.

[17] Dai, S.; Zhao, F.; Zhang, Q.; Lau, T.-K.; Li, T.; Liu, K.; Ling, Q.; Wang, C.; Lu, X.; You, W.; Zhan, X., Fused Nonacyclic Electron Acceptors for Efficient Polymer Solar Cells. *Journal of the American Chemical Society* **2017**, *139 (3)*, 1336-1343.

Author Contributions

C.L., C.I. and Y.Y. contributed equally to this work. C.L. and Y.Y. proposed the research. K.R. designed the CI-TTIN-2F. C.I. performed the synthesis and material characterization. C.L. and Y.Y. performed the solar cell fabrication, measurement and optimization. O.A.S. and M.A.S. carried out the theoretical calculation. H.K. and N.S. conducted and optimized the GIWAXS. B.D. contributed to modules fabrication. X.Z., and V.J. provided technical support on hole mobility analysis. Y.D., S.D. P.D, and M.K.N. directed this work. C.L. C.I. and Y.Y. wrote the first draft of the manuscript. All the authors revised and approved the manuscript.
